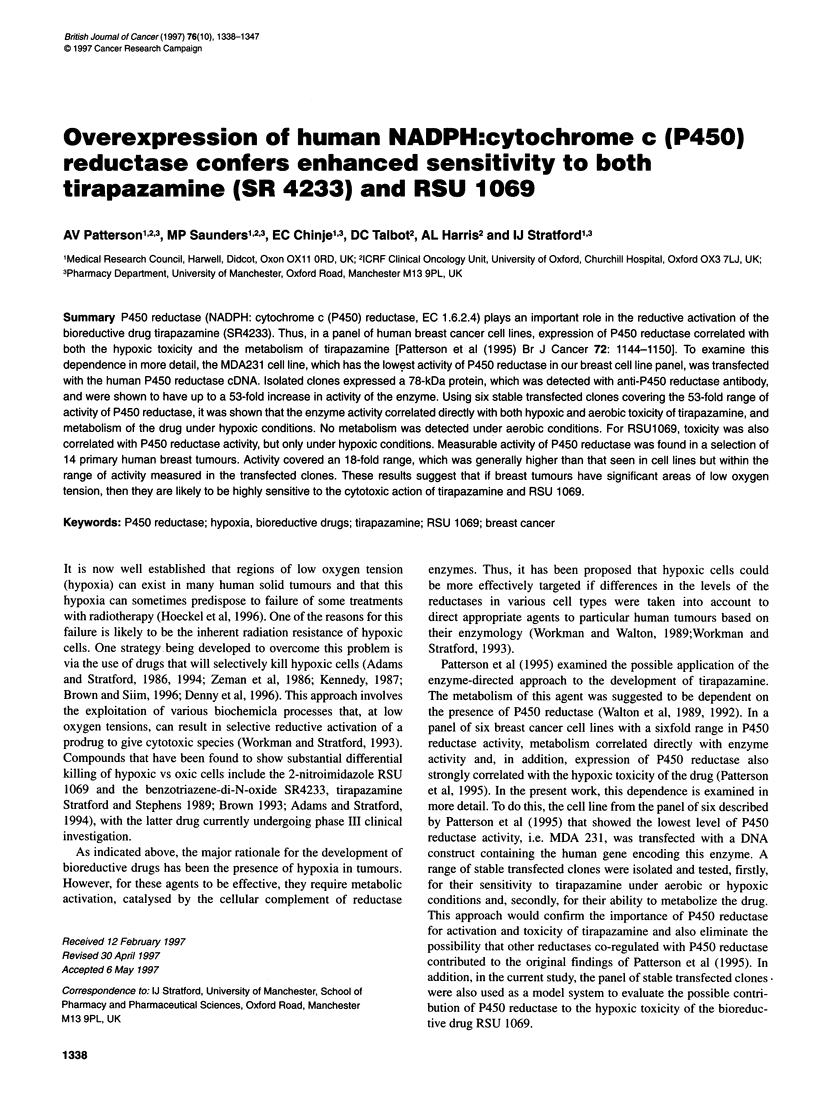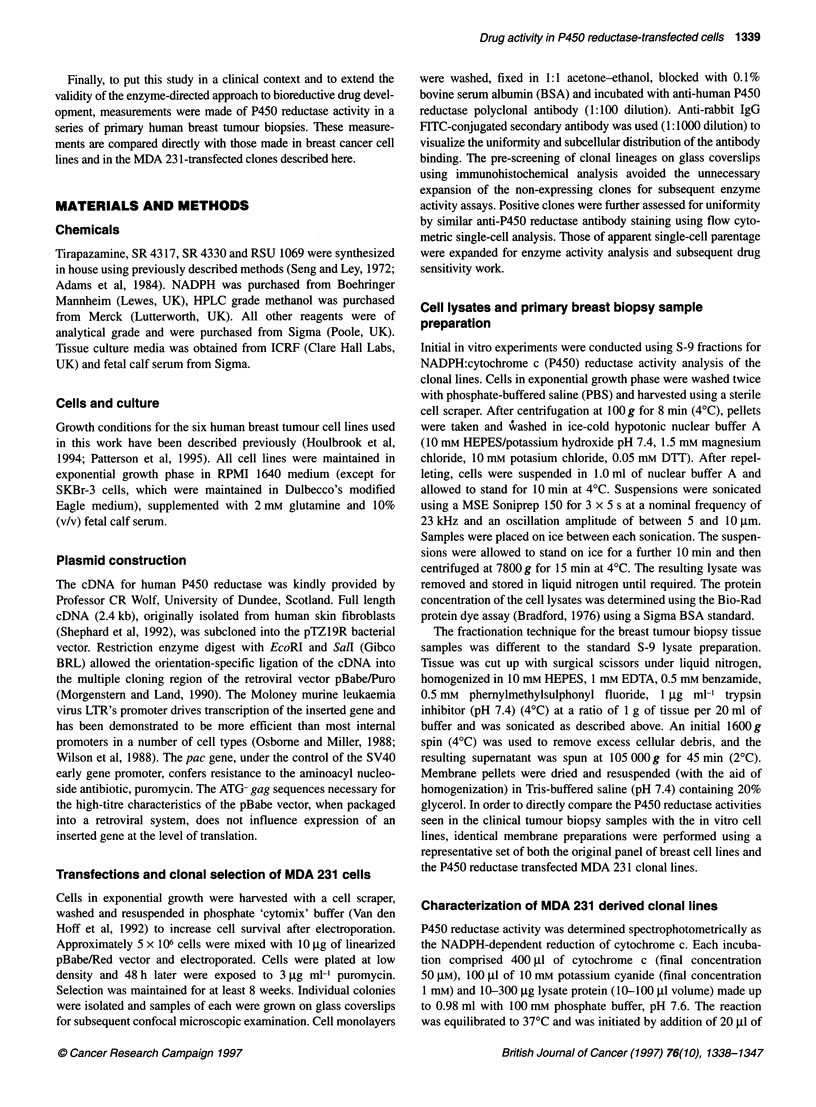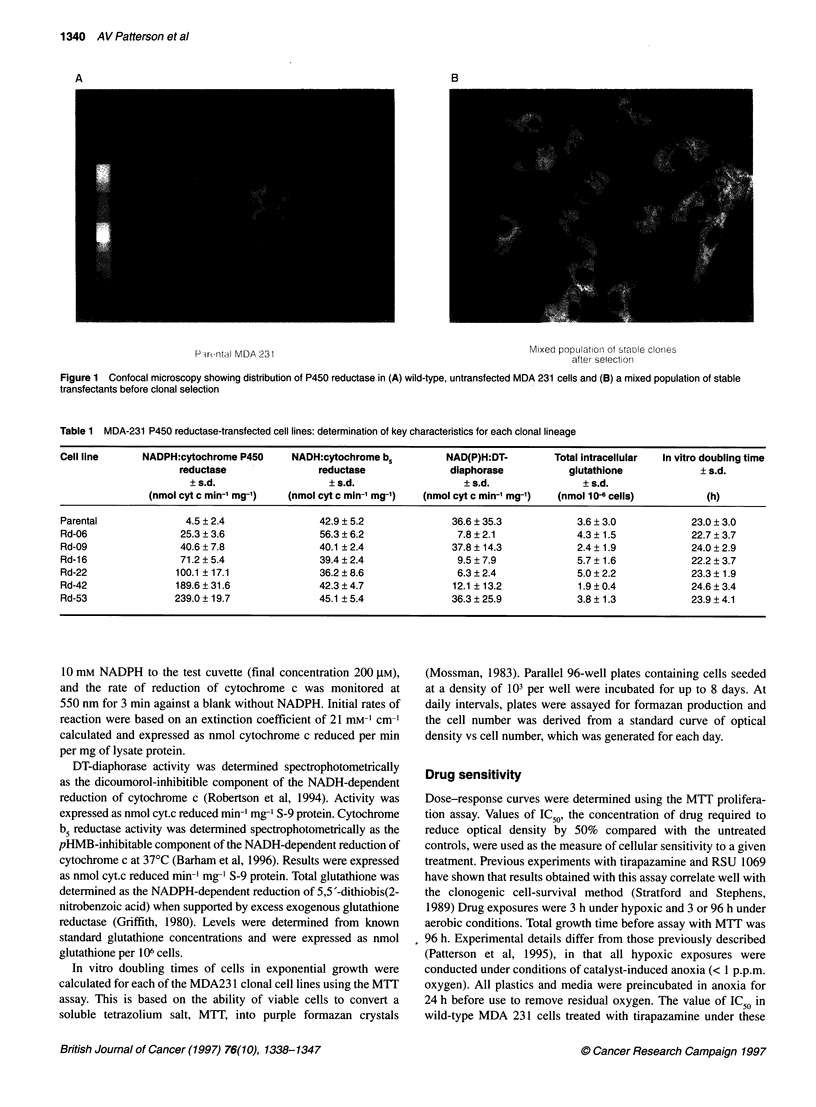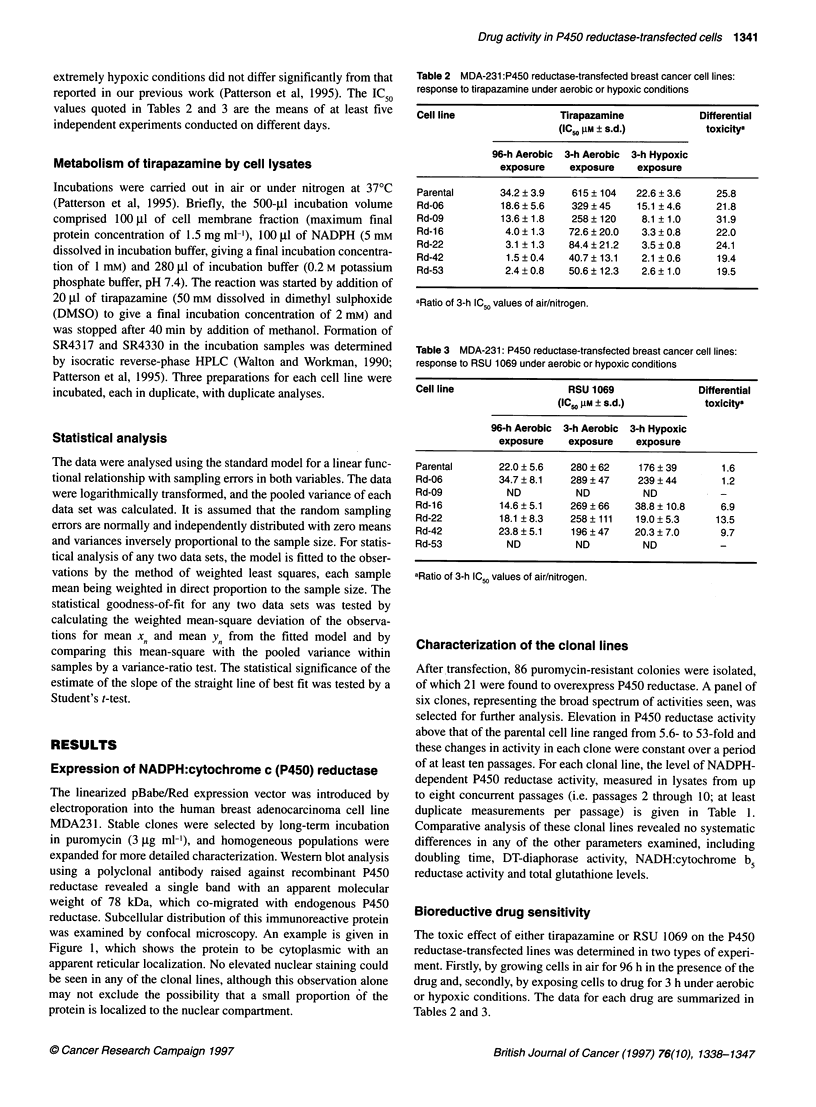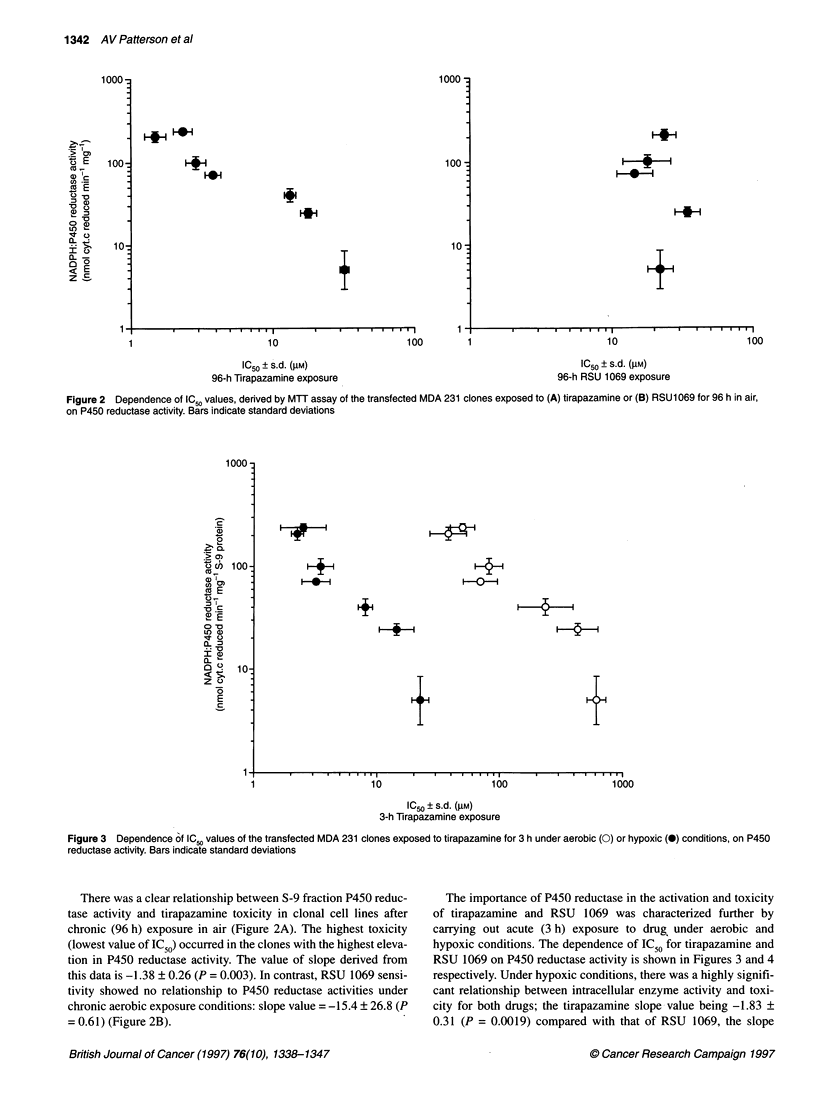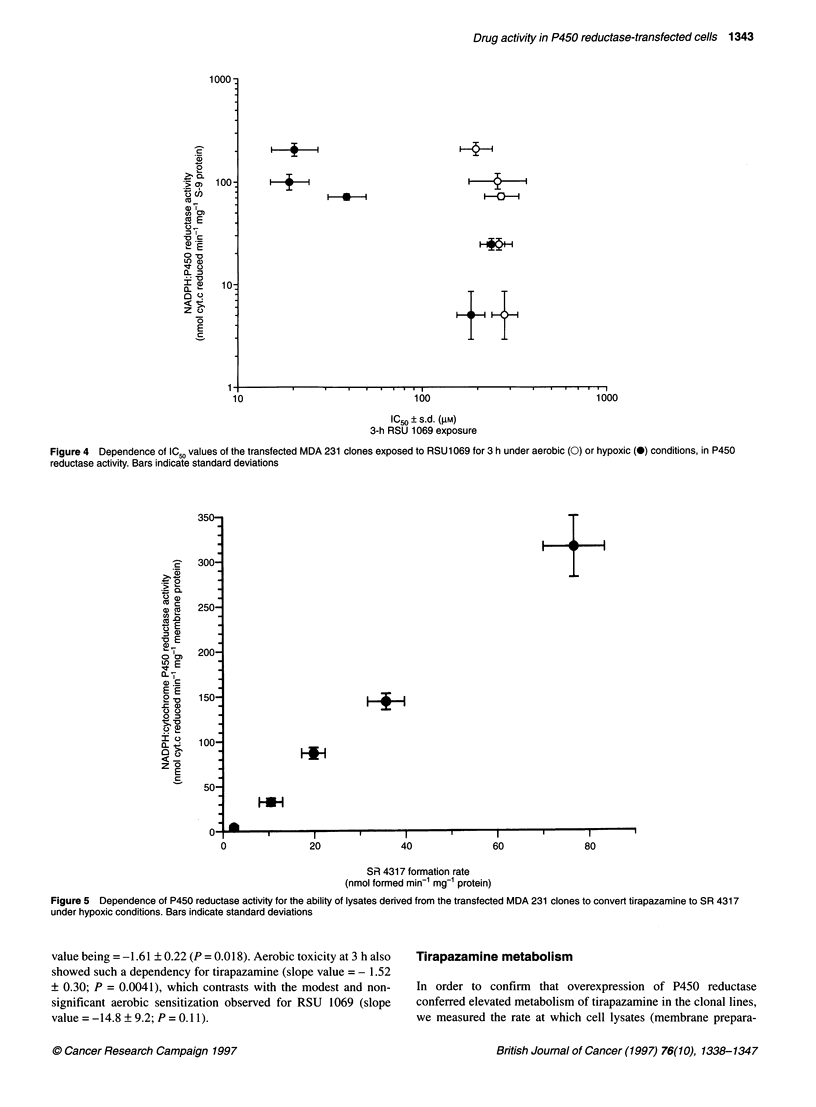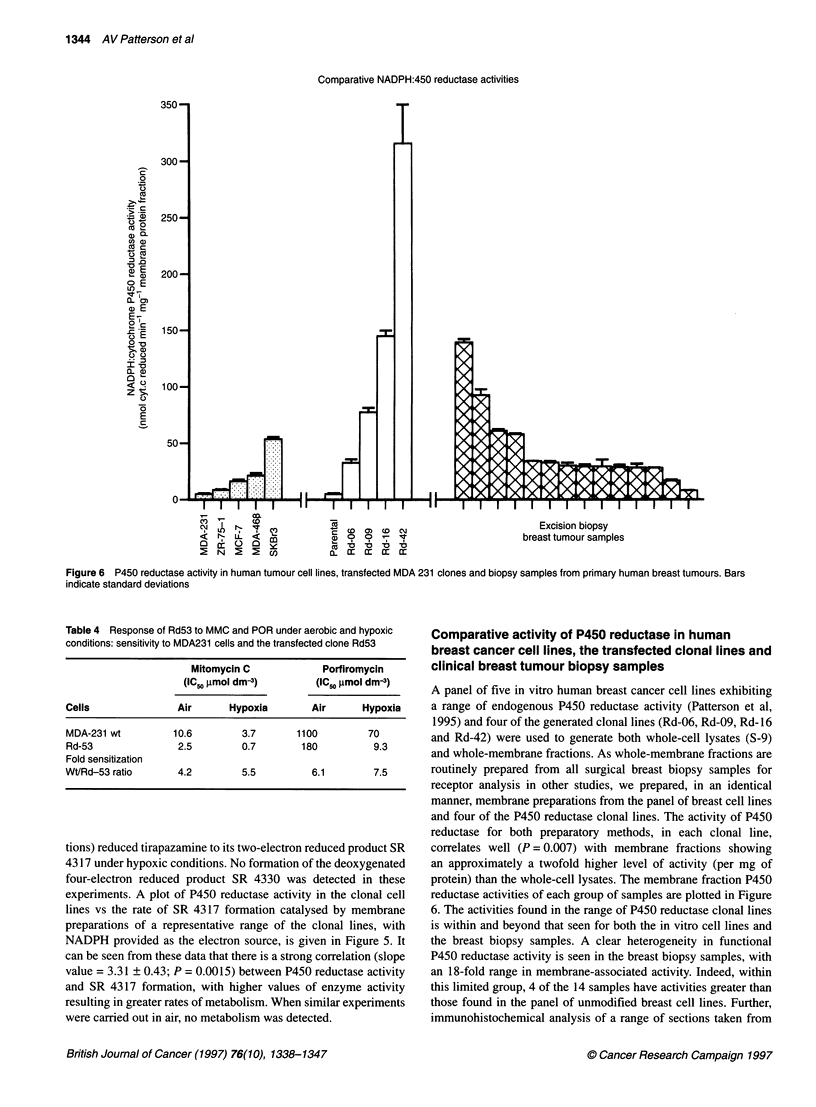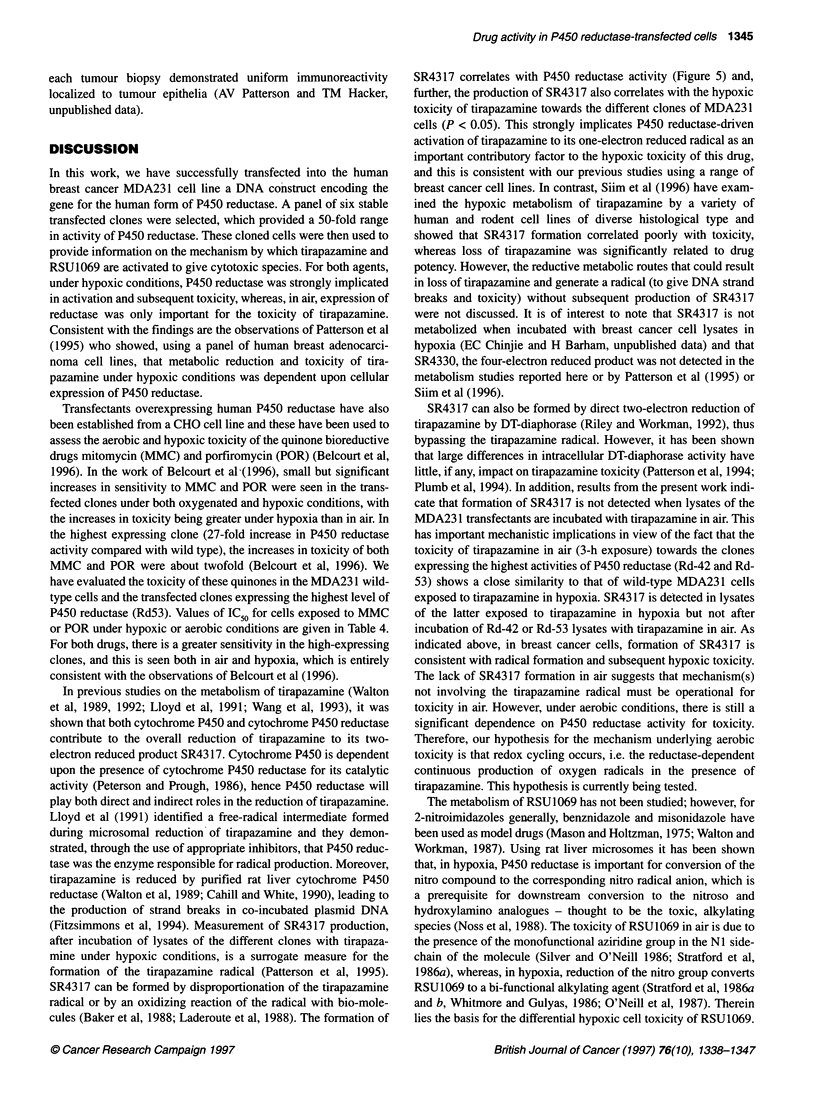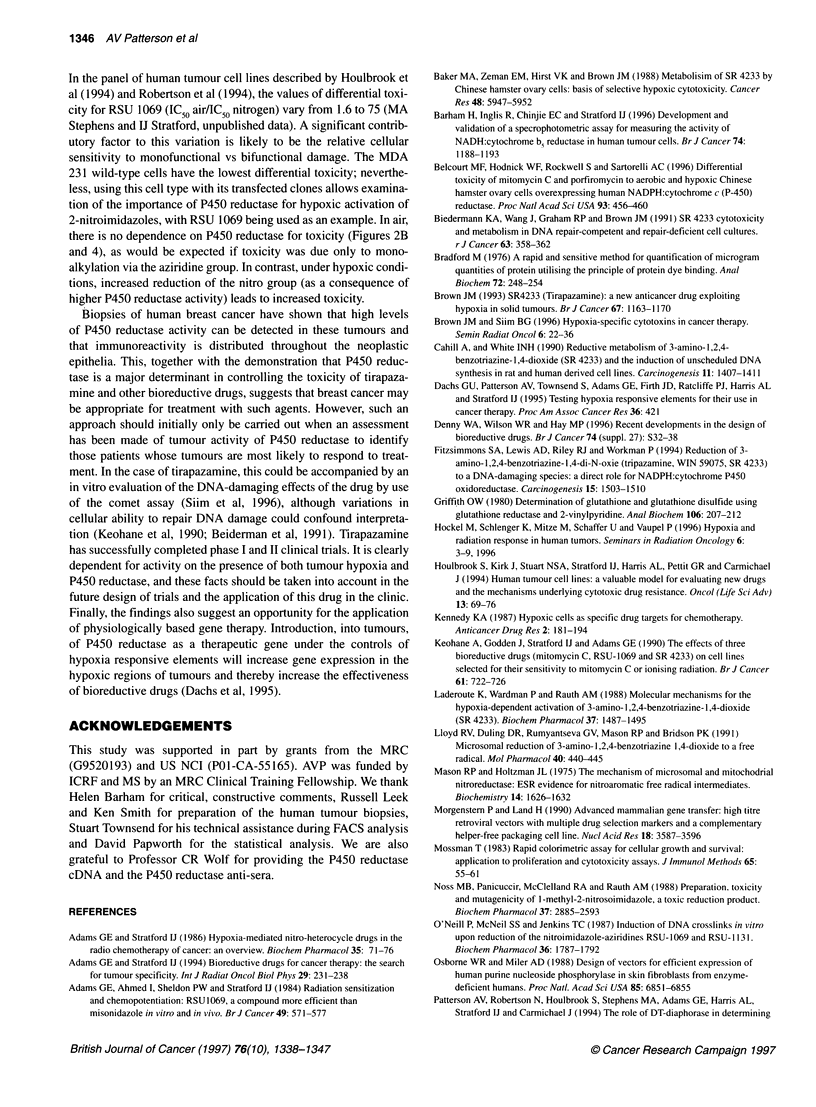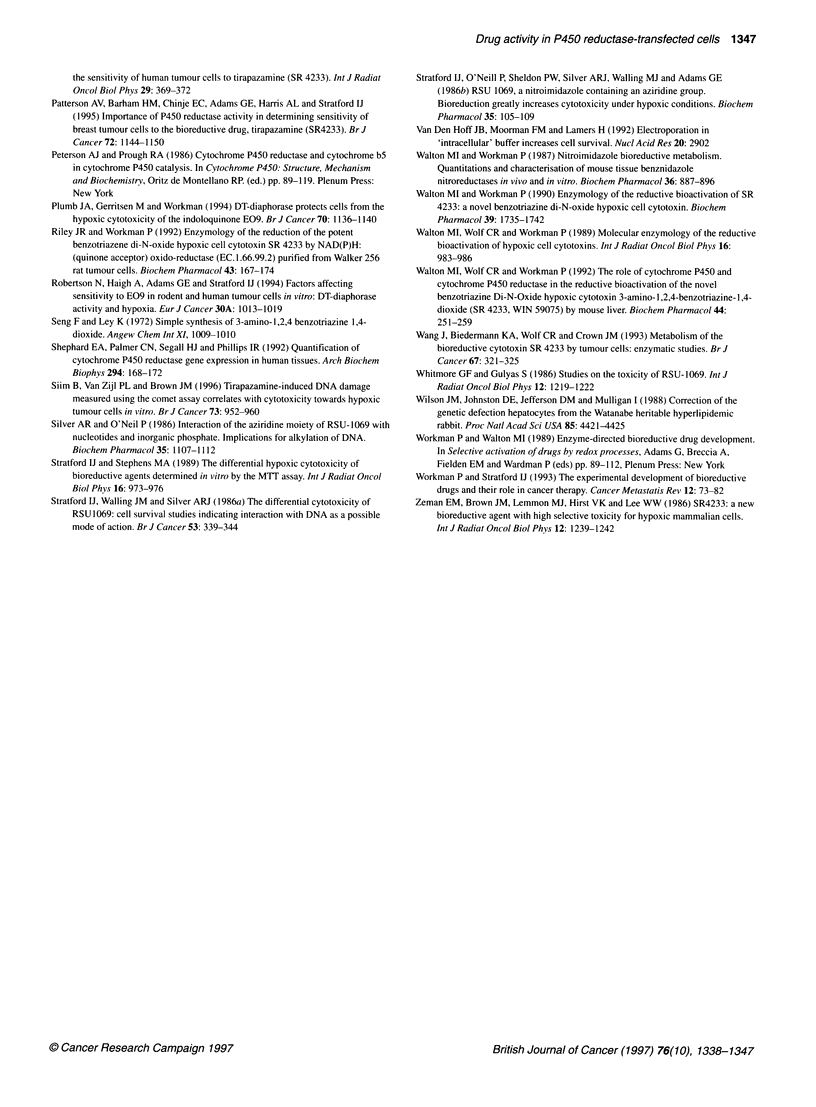# Overexpression of human NADPH:cytochrome c (P450) reductase confers enhanced sensitivity to both tirapazamine (SR 4233) and RSU 1069.

**DOI:** 10.1038/bjc.1997.558

**Published:** 1997

**Authors:** A. V. Patterson, M. P. Saunders, E. C. Chinje, D. C. Talbot, A. L. Harris, I. J. Strafford

**Affiliations:** Medical Research Council, Didcot, Oxon, UK.

## Abstract

**Images:**


British Journal of Cancer (1997) 76(10), 1338–1347
© 1997 Cancer Research Campaign

### Overexpression of human NADPH:cytochrome c (P450) reductase confers enhanced sensitivity to both tirapazamine (SR 4233) and RSU 1069

**AV Patterson[1,2,3], MP Saunders[1,2,3], EC Chinje[1,3], DC Talbot[2], AL Harris[2] and IJ Stratford[1,3]**

[1]Medical Research Council, Harwell, Didcot, Oxon OX11 0RD, UK; [2]ICRF Clinical Oncology Unit, University of Oxford, Churchill Hospital, Oxford OX3 7LJ, UK; [3]Pharmacy Department, University of Manchester, Oxford Road, Manchester M13 9PL, UK

**Summary** P450 reductase (NADPH: cytochrome c (P450) reductase, EC 1.6.2.4) plays an important role in the reductive activation of the bioreductive drug tirapazamine (SR4233). Thus, in a panel of human breast cancer cell lines, expression of P450 reductase correlated with both the hypoxic toxicity and the metabolism of tirapazamine [Patterson et al (1995) Br J Cancer **72**: 1144–1150]. To examine this dependence in more detail, the MDA231 cell line, which has the lowest activity of P450 reductase in our breast cell line panel, was transfected with the human P450 reductase cDNA. Isolated clones expressed a 78-kDa protein, which was detected with anti-P450 reductase antibody, and were shown to have up to a 53-fold increase in activity of the enzyme. Using six stable transfected clones covering the 53-fold range of activity of P450 reductase, it was shown that the enzyme activity correlated directly with both hypoxic and aerobic toxicity of tirapazamine, and metabolism of the drug under hypoxic conditions. No metabolism was detected under aerobic conditions. For RSU1069, toxicity was also correlated with P450 reductase activity, but only under hypoxic conditions. Measurable activity of P450 reductase was found in a selection of 14 primary human breast tumours. Activity covered an 18-fold range, which was generally higher than that seen in cell lines but within the range of activity measured in the transfected clones. These results suggest that if breast tumours have significant areas of low oxygen tension, then they are likely to be highly sensitive to the cytotoxic action of tirapazamine and RSU 1069.

**Keywords:** P450 reductase; hypoxia, bioreductive drugs; tirapazamine; RSU 1069; breast cancer

It is now well established that regions of low oxygen tension (hypoxia) can exist in many human solid tumours and that this hypoxia can sometimes predispose to failure of some treatments with radiotherapy (Hoeckel et al, 1996). One of the reasons for this failure is likely to be the inherent radiation resistance of hypoxic cells. One strategy being developed to overcome this problem is via the use of drugs that will selectively kill hypoxic cells (Adams and Stratford, 1986, 1994; Zeman et al, 1986; Kennedy, 1987; Brown and Siim, 1996; Denny et al, 1996). This approach involves the exploitation of various biochemicla processes that, at low oxygen tensions, can result in selective reductive activation of a prodrug to give cytotoxic species (Workman and Stratford, 1993). Compounds that have been found to show substantial differential killing of hypoxic vs oxic cells include the 2-nitroimidazole RSU 1069 and the benzotriazene-di-N-oxide SR4233, tirapazamine Stratford and Stephens 1989; Brown 1993; Adams and Stratford, 1994), with the latter drug currently undergoing phase III clinical investigation.

As indicated above, the major rationale for the development of bioreductive drugs has been the presence of hypoxia in tumours. However, for these agents to be effective, they require metabolic activation, catalysed by the cellular complement of reductase enzymes. Thus, it has been proposed that hypoxic cells could be more effectively targeted if differences in the levels of the reductases in various cell types were taken into account to direct appropriate agents to particular human tumours based on their enzymology (Workman and Walton, 1989;Workman and Stratford, 1993).

Patterson et al (1995) examined the possible application of the enzyme-directed approach to the development of tirapazamine. The metabolism of this agent was suggested to be dependent on the presence of P450 reductase (Walton et al, 1989, 1992). In a panel of six breast cancer cell lines with a sixfold range in P450 reductase activity, metabolism correlated directly with enzyme activity and, in addition, expression of P450 reductase also strongly correlated with the hypoxic toxicity of the drug (Patterson et al, 1995). In the present work, this dependence is examined in more detail. To do this, the cell line from the panel of six described by Patterson et al (1995) that showed the lowest level of P450 reductase activity, i.e. MDA 231, was transfected with a DNA construct containing the human gene encoding this enzyme. A range of stable transfected clones were isolated and tested, firstly, for their sensitivity to tirapazamine under aerobic or hypoxic conditions and, secondly, for their ability to metabolize the drug. This approach would confirm the importance of P450 reductase for activation and toxicity of tirapazamine and also eliminate the possibility that other reductases co-regulated with P450 reductase contributed to the original findings of Patterson et al (1995). In addition, in the current study, the panel of stable transfected clones were also used as a model system to evaluate the possible contribution of P450 reductase to the hypoxic toxicity of the bioreductive drug RSU 1069.

*Received 12 February 1997*
*Revised 30 April 1997*
*Accepted 6 May 1997*

*Correspondence to:* IJ Stratford, University of Manchester, School of Pharmacy and Pharmaceutical Sciences, Oxford Road, Manchester M13 9PL, UK

Finally, to put this study in a clinical context and to extend the validity of the enzyme-directed approach to bioreductive drug development, measurements were made of P450 reductase activity in a series of primary human breast tumour biopsies. These measurements are compared directly with those made in breast cancer cell lines and in the MDA 231-transfected clones described here.

### MATERIALS AND METHODS

### Chemicals

Tirapazamine, SR 4317, SR 4330 and RSU 1069 were synthesized in house using previously described methods (Seng and Ley, 1972; Adams et al, 1984). NADPH was purchased from Boehringer Mannheim (Lewes, UK), HPLC grade methanol was purchased from Merck (Lutterworth, UK). All other reagents were of analytical grade and were purchased from Sigma (Poole, UK). Tissue culture media was obtained from ICRF (Clare Hall Labs, UK) and fetal calf serum from Sigma.

### Cells and culture

Growth conditions for the six human breast tumour cell lines used in this work have been described previously (Houlbrook et al, 1994; Patterson et al, 1995). All cell lines were maintained in exponential growth phase in RPMI 1640 medium (except for SKBr-3 cells, which were maintained in Dulbecco's modified Eagle medium), supplemented with 2 mM glutamine and 10% (v/v) fetal calf serum.

### Plasmid construction

The cDNA for human P450 reductase was kindly provided by Professor CR Wolf, University of Dundee, Scotland. Full length cDNA (2.4 kb), originally isolated from human skin fibroblasts (Shephard et al, 1992), was subcloned into the pTZ19R bacterial vector. Restriction enzyme digest with *Eco*RI and *Sal*I (Gibco BRL) allowed the orientation-specific ligation of the cDNA into the multiple cloning region of the retroviral vector pBabe/Puro (Morgenstern and Land, 1990). The Moloney murine leukaemia virus LTR's promoter drives transcription of the inserted gene and has been demonstrated to be more efficient than most internal promoters in a number of cell types (Osborne and Miller, 1988; Wilson et al, 1988). The *pac* gene, under the control of the SV40 early gene promoter, confers resistance to the aminoacyl nucleoside antibiotic, puromycin. The ATG$^-$ *gag* sequences necessary for the high-titre characteristics of the pBabe vector, when packaged into a retroviral system, does not influence expression of an inserted gene at the level of translation.

### Transfections and clonal selection of MDA 231 cells

Cells in exponential growth were harvested with a cell scraper, washed and resuspended in phosphate 'cytomix' buffer (Van den Hoff et al, 1992) to increase cell survival after electroporation. Approximately $5 \times 10^6$ cells were mixed with 10 μg of linearized pBabe/Red vector and electroporated. Cells were plated at low density and 48 h later were exposed to 3 μg ml$^{-1}$ puromycin. Selection was maintained for at least 8 weeks. Individual colonies were isolated and samples of each were grown on glass coverslips for subsequent confocal microscopic examination. Cell monolayers were washed, fixed in 1:1 acetone–ethanol, blocked with 0.1% bovine serum albumin (BSA) and incubated with anti-human P450 reductase polyclonal antibody (1:100 dilution). Anti-rabbit IgG FITC-conjugated secondary antibody was used (1:1000 dilution) to visualize the uniformity and subcellular distribution of the antibody binding. The pre-screening of clonal lineages on glass coverslips using immunohistochemical analysis avoided the unnecessary expansion of the non-expressing clones for subsequent enzyme activity assays. Positive clones were further assessed for uniformity by similar anti-P450 reductase antibody staining using flow cytometric single-cell analysis. Those of apparent single-cell parentage were expanded for enzyme activity analysis and subsequent drug sensitivity work.

### Cell lysates and primary breast biopsy sample preparation

Initial in vitro experiments were conducted using S-9 fractions for NADPH:cytochrome c (P450) reductase activity analysis of the clonal lines. Cells in exponential growth phase were washed twice with phosphate-buffered saline (PBS) and harvested using a sterile cell scraper. After centrifugation at 100 *g* for 8 min (4°C), pellets were taken and washed in ice-cold hypotonic nuclear buffer A (10 mM HEPES/potassium hydroxide pH 7.4, 1.5 mM magnesium chloride, 10 mM potasium chloride, 0.05 mM DTT). After repelleting, cells were suspended in 1.0 ml of nuclear buffer A and allowed to stand for 10 min at 4°C. Suspensions were sonicated using a MSE Soniprep 150 for $3 \times 5$ s at a nominal frequency of 23 kHz and an oscillation amplitude of between 5 and 10 μm. Samples were placed on ice between each sonication. The suspensions were allowed to stand on ice for a further 10 min and then centrifuged at 7800 *g* for 15 min at 4°C. The resulting lysate was removed and stored in liquid nitrogen until required. The protein concentration of the cell lysates was determined using the Bio-Rad protein dye assay (Bradford, 1976) using a Sigma BSA standard.

The fractionation technique for the breast tumour biopsy tissue samples was different to the standard S-9 lysate preparation. Tissue was cut up with surgical scissors under liquid nitrogen, homogenized in 10 mM HEPES, 1 mM EDTA, 0.5 mM benzamide, 0.5 mM phernylmethylsulphonyl fluoride, 1 μg ml$^{-1}$ trypsin inhibitor (pH 7.4) (4°C) at a ratio of 1 g of tissue per 20 ml of buffer and was sonicated as described above. An initial 1600 *g* spin (4°C) was used to remove excess cellular debris, and the resulting supernatant was spun at 105 000 *g* for 45 min (2°C). Membrane pellets were dried and resuspended (with the aid of homogenization) in Tris-buffered saline (pH 7.4) containing 20% glycerol. In order to directly compare the P450 reductase activities seen in the clinical tumour biopsy samples with the in vitro cell lines, identical membrane preparations were performed using a representative set of both the original panel of breast cell lines and the P450 reductase transfected MDA 231 clonal lines.

### Characterization of MDA 231 derived clonal lines

P450 reductase activity was determined spectrophotometrically as the NADPH-dependent reduction of cytochrome c. Each incubation comprised 400 μl of cytochrome c (final concentration 50 μM), 100 μl of 10 mM potassium cyanide (final concentration 1 mM) and 10–300 μg lysate protein (10–100 μl volume) made up to 0.98 ml with 100 mM phosphate buffer, pH 7.6. The reaction was equilibrated to 37°C and was initiated by addition of 20 μl of

© Cancer Research Campaign 1997

**Figure 1** Confocal microscopy showing distribution of P450 reductase in (**A**) wild-type, untransfected MDA 231 cells and (**B**) a mixed population of stable transfectants before clonal selection

**Table 1** MDA-231 P450 reductase-transfected cell lines: determination of key characteristics for each clonal lineage

| Cell line | NADPH:cytochrome P450 reductase ± s.d. (nmol cyt c min$^{-1}$ mg$^{-1}$) | NADH:cytochrome $b_5$ reductase ± s.d. (nmol cyt c min$^{-1}$ mg$^{-1}$) | NAD(P)H:DT-diaphorase ± s.d. (nmol cyt c min$^{-1}$ mg$^{-1}$) | Total intracellular glutathione ± s.d. (nmol $10^{-6}$ cells) | In vitro doubling time ± s.d. (h) |
|---|---|---|---|---|---|
| Parental | 4.5 ± 2.4 | 42.9 ± 5.2 | 36.6 ± 35.3 | 3.6 ± 3.0 | 23.0 ± 3.0 |
| Rd-06 | 25.3 ± 3.6 | 56.3 ± 6.2 | 7.8 ± 2.1 | 4.3 ± 1.5 | 22.7 ± 3.7 |
| Rd-09 | 40.6 ± 7.8 | 40.1 ± 2.4 | 37.8 ± 14.3 | 2.4 ± 1.9 | 24.0 ± 2.9 |
| Rd-16 | 71.2 ± 5.4 | 39.4 ± 2.4 | 9.5 ± 7.9 | 5.7 ± 1.6 | 22.2 ± 3.7 |
| Rd-22 | 100.1 ± 17.1 | 36.2 ± 8.6 | 6.3 ± 2.4 | 5.0 ± 2.2 | 23.3 ± 1.9 |
| Rd-42 | 189.6 ± 31.6 | 42.3 ± 4.7 | 12.1 ± 13.2 | 1.9 ± 0.4 | 24.6 ± 3.4 |
| Rd-53 | 239.0 ± 19.7 | 45.1 ± 5.4 | 36.3 ± 25.9 | 3.8 ± 1.3 | 23.9 ± 4.1 |

10 mM NADPH to the test cuvette (final concentration 200 μM), and the rate of reduction of cytochrome c was monitored at 550 nm for 3 min against a blank without NADPH. Initial rates of reaction were based on an extinction coefficient of 21 mM$^{-1}$ cm$^{-1}$ calculated and expressed as nmol cytochrome c reduced per min per mg of lysate protein.

DT-diaphorase activity was determined spectrophotometrically as the dicoumorol-inhibitible component of the NADH-dependent reduction of cytochrome c (Robertson et al, 1994). Activity was expressed as nmol cyt.c reduced min$^{-1}$ mg$^{-1}$ S-9 protein. Cytochrome $b_5$ reductase activity was determined spectrophotometrically as the *p*HMB-inhibitable component of the NADH-dependent reduction of cytochrome c at 37°C (Barham et al, 1996). Results were expressed as nmol cyt.c reduced min$^{-1}$ mg$^{-1}$ S-9 protein. Total glutathione was determined as the NADPH-dependent reduction of 5,5′-dithiobis(2-nitrobenzoic acid) when supported by excess exogenous glutathione reductase (Griffith, 1980). Levels were determined from known standard glutathione concentrations and were expressed as nmol glutathione per $10^6$ cells.

In vitro doubling times of cells in exponential growth were calculated for each of the MDA231 clonal cell lines using the MTT assay. This is based on the ability of viable cells to convert a soluble tetrazolium salt, MTT, into purple formazan crystals (Mossman, 1983). Parallel 96-well plates containing cells seeded at a density of $10^3$ per well were incubated for up to 8 days. At daily intervals, plates were assayed for formazan production and the cell number was derived from a standard curve of optical density vs cell number, which was generated for each day.

### Drug sensitivity

Dose–response curves were determined using the MTT proliferation assay. Values of $IC_{50}$, the concentration of drug required to reduce optical density by 50% compared with the untreated controls, were used as the measure of cellular sensitivity to a given treatment. Previous experiments with tirapazamine and RSU 1069 have shown that results obtained with this assay correlate well with the clonogenic cell-survival method (Stratford and Stephens, 1989) Drug exposures were 3 h under hypoxic and 3 or 96 h under aerobic conditions. Total growth time before assay with MTT was 96 h. Experimental details differ from those previously described (Patterson et al, 1995), in that all hypoxic exposures were conducted under conditions of catalyst-induced anoxia (< 1 p.p.m. oxygen). All plastics and media were preincubated in anoxia for 24 h before use to remove residual oxygen. The value of $IC_{50}$ in wild-type MDA 231 cells treated with tirapazamine under these

© Cancer Research Campaign 1997

extremely hypoxic conditions did not differ significantly from that reported in our previous work (Patterson et al, 1995). The $IC_{50}$ values quoted in Tables 2 and 3 are the means of at least five independent experiments conducted on different days.

### Metabolism of tirapazamine by cell lysates

Incubations were carried out in air or under nitrogen at 37°C (Patterson et al, 1995). Briefly, the 500-µl incubation volume comprised 100 µl of cell membrane fraction (maximum final protein concentration of 1.5 mg $ml^{-1}$), 100 µl of NADPH (5 mM dissolved in incubation buffer, giving a final incubation concentration of 1 mM) and 280 µl of incubation buffer (0.2 M potassium phosphate buffer, pH 7.4). The reaction was started by addition of 20 µl of tirapazamine (50 mM dissolved in dimethyl sulphoxide (DMSO) to give a final incubation concentration of 2 mM) and was stopped after 40 min by addition of methanol. Formation of SR4317 and SR4330 in the incubation samples was determined by isocratic reverse-phase HPLC (Walton and Workman, 1990; Patterson et al, 1995). Three preparations for each cell line were incubated, each in duplicate, with duplicate analyses.

### Statistical analysis

The data were analysed using the standard model for a linear functional relationship with sampling errors in both variables. The data were logarithmically transformed, and the pooled variance of each data set was calculated. It is assumed that the random sampling errors are normally and independently distributed with zero means and variances inversely proportional to the sample size. For statistical analysis of any two data sets, the model is fitted to the observations by the method of weighted least squares, each sample mean being weighted in direct proportion to the sample size. The statistical goodness-of-fit for any two data sets was tested by calculating the weighted mean-square deviation of the observations for mean $x_n$ and mean $y_n$ from the fitted model and by comparing this mean-square with the pooled variance within samples by a variance-ratio test. The statistical significance of the estimate of the slope of the straight line of best fit was tested by a Student's *t*-test.

### RESULTS

### Expression of NADPH:cytochrome c (P450) reductase

The linearized pBabe/Red expression vector was introduced by electroporation into the human breast adenocarcinoma cell line MDA231. Stable clones were selected by long-term incubation in puromycin (3 µg $ml^{-1}$), and homogeneous populations were expanded for more detailed characterization. Western blot analysis using a polyclonal antibody raised against recombinant P450 reductase revealed a single band with an apparent molecular weight of 78 kDa, which co-migrated with endogenous P450 reductase. Subcellular distribution of this immunoreactive protein was examined by confocal microscopy. An example is given in Figure 1, which shows the protein to be cytoplasmic with an apparent reticular localization. No elevated nuclear staining could be seen in any of the clonal lines, although this observation alone may not exclude the possibility that a small proportion of the protein is localized to the nuclear compartment.

**Table 2** MDA-231:P450 reductase-transfected breast cancer cell lines: response to tirapazamine under aerobic or hypoxic conditions

| Cell line | Tirapazamine ($IC_{50}$ µM ± s.d.) | | | Differential toxicity[a] |
|---|---|---|---|---|
| | 96-h Aerobic exposure | 3-h Aerobic exposure | 3-h Hypoxic exposure | |
| Parental | 34.2 ± 3.9 | 615 ± 104 | 22.6 ± 3.6 | 25.8 |
| Rd-06 | 18.6 ± 5.6 | 329 ± 45 | 15.1 ± 4.6 | 21.8 |
| Rd-09 | 13.6 ± 1.8 | 258 ± 120 | 8.1 ± 1.0 | 31.9 |
| Rd-16 | 4.0 ± 1.3 | 72.6 ± 20.0 | 3.3 ± 0.8 | 22.0 |
| Rd-22 | 3.1 ± 1.3 | 84.4 ± 21.2 | 3.5 ± 0.8 | 24.1 |
| Rd-42 | 1.5 ± 0.4 | 40.7 ± 13.1 | 2.1 ± 0.6 | 19.4 |
| Rd-53 | 2.4 ± 0.8 | 50.6 ± 12.3 | 2.6 ± 1.0 | 19.5 |

[a]Ratio of 3-h $IC_{50}$ values of air/nitrogen.

**Table 3** MDA-231: P450 reductase-transfected breast cancer cell lines: response to RSU 1069 under aerobic or hypoxic conditions

| Cell line | RSU 1069 ($IC_{50}$ µM ± s.d.) | | | Differential toxicity[a] |
|---|---|---|---|---|
| | 96-h Aerobic exposure | 3-h Aerobic exposure | 3-h Hypoxic exposure | |
| Parental | 22.0 ± 5.6 | 280 ± 62 | 176 ± 39 | 1.6 |
| Rd-06 | 34.7 ± 8.1 | 289 ± 47 | 239 ± 44 | 1.2 |
| Rd-09 | ND | ND | ND | – |
| Rd-16 | 14.6 ± 5.1 | 269 ± 66 | 38.8 ± 10.8 | 6.9 |
| Rd-22 | 18.1 ± 8.3 | 258 ± 111 | 19.0 ± 5.3 | 13.5 |
| Rd-42 | 23.8 ± 5.1 | 196 ± 47 | 20.3 ± 7.0 | 9.7 |
| Rd-53 | ND | ND | ND | – |

[a]Ratio of 3-h $IC_{50}$ values of air/nitrogen.

### Characterization of the clonal lines

After transfection, 86 puromycin-resistant colonies were isolated, of which 21 were found to overexpress P450 reductase. A panel of six clones, representing the broad spectrum of activities seen, was selected for further analysis. Elevation in P450 reductase activity above that of the parental cell line ranged from 5.6- to 53-fold and these changes in activity in each clone were constant over a period of at least ten passages. For each clonal line, the level of NADPH-dependent P450 reductase activity, measured in lysates from up to eight concurrent passages (i.e. passages 2 through 10; at least duplicate measurements per passage) is given in Table 1. Comparative analysis of these clonal lines revealed no systematic differences in any of the other parameters examined, including doubling time, DT-diaphorase activity, NADH:cytochrome $b_5$ reductase activity and total glutathione levels.

### Bioreductive drug sensitivity

The toxic effect of either tirapazamine or RSU 1069 on the P450 reductase-transfected lines was determined in two types of experiment. Firstly, by growing cells in air for 96 h in the presence of the drug and, secondly, by exposing cells to drug for 3 h under aerobic or hypoxic conditions. The data for each drug are summarized in Tables 2 and 3.

© Cancer Research Campaign 1997

**Figure 2** Dependence of $IC_{50}$ values, derived by MTT assay of the transfected MDA 231 clones exposed to (**A**) tirapazamine or (**B**) RSU1069 for 96 h in air, on P450 reductase activity. Bars indicate standard deviations

**Figure 3** Dependence of $IC_{50}$ values of the transfected MDA 231 clones exposed to tirapazamine for 3 h under aerobic (○) or hypoxic (●) conditions, on P450 reductase activity. Bars indicate standard deviations

There was a clear relationship between S-9 fraction P450 reductase activity and tirapazamine toxicity in clonal cell lines after chronic (96 h) exposure in air (Figure 2A). The highest toxicity (lowest value of $IC_{50}$) occurred in the clones with the highest elevation in P450 reductase activity. The value of slope derived from this data is –1.38 ± 0.26 ($P$ = 0.003). In contrast, RSU 1069 sensitivity showed no relationship to P450 reductase activities under chronic aerobic exposure conditions: slope value = –15.4 ± 26.8 ($P$ = 0.61) (Figure 2B).

The importance of P450 reductase in the activation and toxicity of tirapazamine and RSU 1069 was characterized further by carrying out acute (3 h) exposure to drug under aerobic and hypoxic conditions. The dependence of $IC_{50}$ for tirapazamine and RSU 1069 on P450 reductase activity is shown in Figures 3 and 4 respectively. Under hypoxic conditions, there was a highly significant relationship between intracellular enzyme activity and toxicity for both drugs; the tirapazamine slope value being –1.83 ± 0.31 ($P$ = 0.0019) compared with that of RSU 1069, the slope

© Cancer Research Campaign 1997

**Figure 4** Dependence of $IC_{50}$ values of the transfected MDA 231 clones exposed to RSU1069 for 3 h under aerobic (○) or hypoxic (●) conditions, in P450 reductase activity. Bars indicate standard deviations

**Figure 5** Dependence of P450 reductase activity for the ability of lysates derived from the transfected MDA 231 clones to convert tirapazamine to SR 4317 under hypoxic conditions. Bars indicate standard deviations value being = −1.61 ± 0.22 ($P$ = 0.018). Aerobic toxicity at 3 h also showed such a dependency for tirapazamine (slope value = − 1.52 ± 0.30; $P$ = 0.0041), which contrasts with the modest and non-significant aerobic sensitization observed for RSU 1069 (slope value = −14.8 ± 9.2; $P$ = 0.11).

### Tirapazamine metabolism

In order to confirm that overexpression of P450 reductase conferred elevated metabolism of tirapazamine in the clonal lines, we measured the rate at which cell lysates (membrane prepara-

© Cancer Research Campaign 1997 

**Figure 6** P450 reductase activity in human tumour cell lines, transfected MDA 231 clones and biopsy samples from primary human breast tumours. Bars indicate standard deviations

**Table 4** Response of Rd53 to MMC and POR under aerobic and hypoxic conditions: sensitivity to MDA231 cells and the transfected clone Rd53

| | Mitomycin C ($IC_{50}$ μmol $dm^{-3}$) | | Porfiromycin ($IC_{50}$ μmol $dm^{-3}$) | |
|---|---|---|---|---|
| **Cells** | **Air** | **Hypoxia** | **Air** | **Hypoxia** |
| MDA-231 wt | 10.6 | 3.7 | 1100 | 70 |
| Rd-53 | 2.5 | 0.7 | 180 | 9.3 |
| Fold sensitization | | | | |
| Wt/Rd–53 ratio | 4.2 | 5.5 | 6.1 | 7.5 |

tions) reduced tirapazamine to its two-electron reduced product SR 4317 under hypoxic conditions. No formation of the deoxygenated four-electron reduced product SR 4330 was detected in these experiments. A plot of P450 reductase activity in the clonal cell lines vs the rate of SR 4317 formation catalysed by membrane preparations of a representative range of the clonal lines, with NADPH provided as the electron source, is given in Figure 5. It can be seen from these data that there is a strong correlation (slope value = 3.31 ± 0.43; $P = 0.0015$) between P450 reductase activity and SR 4317 formation, with higher values of enzyme activity resulting in greater rates of metabolism. When similar experiments were carried out in air, no metabolism was detected.

### Comparative activity of P450 reductase in human breast cancer cell lines, the transfected clonal lines and clinical breast tumour biopsy samples

A panel of five in vitro human breast cancer cell lines exhibiting a range of endogenous P450 reductase activity (Patterson et al, 1995) and four of the generated clonal lines (Rd-06, Rd-09, Rd-16 and Rd-42) were used to generate both whole-cell lysates (S-9) and whole-membrane fractions. As whole-membrane fractions are routinely prepared from all surgical breast biopsy samples for receptor analysis in other studies, we prepared, in an identical manner, membrane preparations from the panel of breast cell lines and four of the P450 reductase clonal lines. The activity of P450 reductase for both preparatory methods, in each clonal line, correlates well ($P = 0.007$) with membrane fractions showing an approximately a twofold higher level of activity (per mg of protein) than the whole-cell lysates. The membrane fraction P450 reductase activities of each group of samples are plotted in Figure 6. The activities found in the range of P450 reductase clonal lines is within and beyond that seen for both the in vitro cell lines and the breast biopsy samples. A clear heterogeneity in functional P450 reductase activity is seen in the breast biopsy samples, with an 18-fold range in membrane-associated activity. Indeed, within this limited group, 4 of the 14 samples have activities greater than those found in the panel of unmodified breast cell lines. Further, immunohistochemical analysis of a range of sections taken from

 © Cancer Research Campaign 1997

each tumour biopsy demonstrated uniform immunoreactivity localized to tumour epithelia (AV Patterson and TM Hacker, unpublished data).

### DISCUSSION

In this work, we have successfully transfected into the human breast cancer MDA231 cell line a DNA construct encoding the gene for the human form of P450 reductase. A panel of six stable transfected clones were selected, which provided a 50-fold range in activity of P450 reductase. These cloned cells were then used to provide information on the mechanism by which tirapazamine and RSU1069 are activated to give cytotoxic species. For both agents, under hypoxic conditions, P450 reductase was strongly implicated in activation and subsequent toxicity, whereas, in air, expression of reductase was only important for the toxicity of tirapazamine. Consistent with the findings are the observations of Patterson et al (1995) who showed, using a panel of human breast adenocarcinoma cell lines, that metabolic reduction and toxicity of tirapazamine under hypoxic conditions was dependent upon cellular expression of P450 reductase.

Transfectants overexpressing human P450 reductase have also been established from a CHO cell line and these have been used to assess the aerobic and hypoxic toxicity of the quinone bioreductive drugs mitomycin (MMC) and porfiromycin (POR) (Belcourt et al, 1996). In the work of Belcourt et al (1996), small but significant increases in sensitivity to MMC and POR were seen in the transfected clones under both oxygenated and hypoxic conditions, with the increases in toxicity being greater under hypoxia than in air. In the highest expressing clone (27-fold increase in P450 reductase activity compared with wild type), the increases in toxicity of both MMC and POR were about twofold (Belcourt et al, 1996). We have evaluated the toxicity of these quinones in the MDA231 wild-type cells and the transfected clones expressing the highest level of P450 reductase (Rd53). Values of $IC_{50}$ for cells exposed to MMC or POR under hypoxic or aerobic conditions are given in Table 4. For both drugs, there is a greater sensitivity in the high-expressing clones, and this is seen both in air and hypoxia, which is entirely consistent with the observations of Belcourt et al (1996).

In previous studies on the metabolism of tirapazamine (Walton et al, 1989, 1992; Lloyd et al, 1991; Wang et al, 1993), it was shown that both cytochrome P450 and cytochrome P450 reductase contribute to the overall reduction of tirapazamine to its two-electron reduced product SR4317. Cytochrome P450 is dependent upon the presence of cytochrome P450 reductase for its catalytic activity (Peterson and Prough, 1986), hence P450 reductase will play both direct and indirect roles in the reduction of tirapazamine. Lloyd et al (1991) identified a free-radical intermediate formed during microsomal reduction of tirapazamine and they demonstrated, through the use of appropriate inhibitors, that P450 reductase was the enzyme responsible for radical production. Moreover, tirapazamine is reduced by purified rat liver cytochrome P450 reductase (Walton et al, 1989; Cahill and White, 1990), leading to the production of strand breaks in co-incubated plasmid DNA (Fitzsimmons et al, 1994). Measurement of SR4317 production, after incubation of lysates of the different clones with tirapazamine under hypoxic conditions, is a surrogate measure for the formation of the tirapazamine radical (Patterson et al, 1995). SR4317 can be formed by disproportionation of the tirapazamine radical or by an oxidizing reaction of the radical with bio-molecules (Baker et al, 1988; Laderoute et al, 1988). The formation of SR4317 correlates with P450 reductase activity (Figure 5) and, further, the production of SR4317 also correlates with the hypoxic toxicity of tirapazamine towards the different clones of MDA231 cells ($P < 0.05$). This strongly implicates P450 reductase-driven activation of tirapazamine to its one-electron reduced radical as an important contributory factor to the hypoxic toxicity of this drug, and this is consistent with our previous studies using a range of breast cancer cell lines. In contrast, Siim et al (1996) have examined the hypoxic metabolism of tirapazamine by a variety of human and rodent cell lines of diverse histological type and showed that SR4317 formation correlated poorly with toxicity, whereas loss of tirapazamine was significantly related to drug potency. However, the reductive metabolic routes that could result in loss of tirapazamine and generate a radical (to give DNA strand breaks and toxicity) without subsequent production of SR4317 were not discussed. It is of interest to note that SR4317 is not metabolized when incubated with breast cancer cell lysates in hypoxia (EC Chinjie and H Barham, unpublished data) and that SR4330, the four-electron reduced product was not detected in the metabolism studies reported here or by Patterson et al (1995) or Siim et al (1996).

SR4317 can also be formed by direct two-electron reduction of tirapazamine by DT-diaphorase (Riley and Workman, 1992), thus bypassing the tirapazamine radical. However, it has been shown that large differences in intracellular DT-diaphorase activity have little, if any, impact on tirapazamine toxicity (Patterson et al, 1994; Plumb et al, 1994). In addition, results from the present work indicate that formation of SR4317 is not detected when lysates of the MDA231 transfectants are incubated with tirapazamine in air. This has important mechanistic implications in view of the fact that the toxicity of tirapazamine in air (3-h exposure) towards the clones expressing the highest activities of P450 reductase (Rd-42 and Rd-53) shows a close similarity to that of wild-type MDA231 cells exposed to tirapazamine in hypoxia. SR4317 is detected in lysates of the latter exposed to tirapazamine in hypoxia but not after incubation of Rd-42 or Rd-53 lysates with tirapazamine in air. As indicated above, in breast cancer cells, formation of SR4317 is consistent with radical formation and subsequent hypoxic toxicity. The lack of SR4317 formation in air suggests that mechanism(s) not involving the tirapazamine radical must be operational for toxicity in air. However, under aerobic conditions, there is still a significant dependence on P450 reductase activity for toxicity. Therefore, our hypothesis for the mechanism underlying aerobic toxicity is that redox cycling occurs, i.e. the reductase-dependent continuous production of oxygen radicals in the presence of tirapazamine. This hypothesis is currently being tested.

The metabolism of RSU1069 has not been studied; however, for 2-nitroimidazoles generally, benznidazole and misonidazole have been used as model drugs (Mason and Holtzman, 1975; Walton and Workman, 1987). Using rat liver microsomes it has been shown that, in hypoxia, P450 reductase is important for conversion of the nitro compound to the corresponding nitro radical anion, which is a prerequisite for downstream conversion to the nitroso and hydroxylamino analogues – thought to be the toxic, alkylating species (Noss et al, 1988). The toxicity of RSU1069 in air is due to the presence of the monofunctional aziridine group in the N1 side-chain of the molecule (Silver and O'Neill 1986; Stratford et al, 1986*a*), whereas, in hypoxia, reduction of the nitro group converts RSU1069 to a bi-functional alkylating agent (Stratford et al, 1986*a* and *b*, Whitmore and Gulyas, 1986; O'Neill et al, 1987). Therein lies the basis for the differential hypoxic cell toxicity of RSU1069.

© Cancer Research Campaign 1997

In the panel of human tumour cell lines described by Houlbrook et al (1994) and Robertson et al (1994), the values of differential toxicity for RSU 1069 ($IC_{50}$ air/$IC_{50}$ nitrogen) vary from 1.6 to 75 (MA Stephens and IJ Stratford, unpublished data). A significant contributory factor to this variation is likely to be the relative cellular sensitivity to monofunctional vs bifunctional damage. The MDA 231 wild-type cells have the lowest differential toxicity; nevertheless, using this cell type with its transfected clones allows examination of the importance of P450 reductase for hypoxic activation of 2-nitroimidazoles, with RSU 1069 being used as an example. In air, there is no dependence on P450 reductase for toxicity (Figures 2B and 4), as would be expected if toxicity was due only to monoalkylation via the aziridine group. In contrast, under hypoxic conditions, increased reduction of the nitro group (as a consequence of higher P450 reductase activity) leads to increased toxicity.

Biopsies of human breast cancer have shown that high levels of P450 reductase activity can be detected in these tumours and that immunoreactivity is distributed throughout the neoplastic epithelia. This, together with the demonstration that P450 reductase is a major determinant in controlling the toxicity of tirapazamine and other bioreductive drugs, suggests that breast cancer may be appropriate for treatment with such agents. However, such an approach should initially only be carried out when an assessment has been made of tumour activity of P450 reductase to identify those patients whose tumours are most likely to respond to treatment. In the case of tirapazamine, this could be accompanied by an in vitro evaluation of the DNA-damaging effects of the drug by use of the comet assay (Siim et al, 1996), although variations in cellular ability to repair DNA damage could confound interpretation (Keohane et al, 1990; Beiderman et al, 1991). Tirapazamine has successfully completed phase I and II clinical trials. It is clearly dependent for activity on the presence of both tumour hypoxia and P450 reductase, and these facts should be taken into account in the future design of trials and the application of this drug in the clinic. Finally, the findings also suggest an opportunity for the application of physiologically based gene therapy. Introduction, into tumours, of P450 reductase as a therapeutic gene under the controls of hypoxia responsive elements will increase gene expression in the hypoxic regions of tumours and thereby increase the effectiveness of bioreductive drugs (Dachs et al, 1995).

### ACKNOWLEDGEMENTS

This study was supported in part by grants from the MRC (G9520193) and US NCI (P01-CA-55165). AVP was funded by ICRF and MS by an MRC Clinical Training Fellowship. We thank Helen Barham for critical, constructive comments, Russell Leek and Ken Smith for preparation of the human tumour biopsies, Stuart Townsend for his technical assistance during FACS analysis and David Papworth for the statistical analysis. We are also grateful to Professor CR Wolf for providing the P450 reductase cDNA and the P450 reductase anti-sera.

### REFERENCES

Adams GE and Stratford IJ (1986) Hypoxia-mediated nitro-heterocycle drugs in the radio chemotherapy of cancer: an overview. *Biochem Pharmacol* **35**: 71–76

Adams GE and Stratford IJ (1994) Bioreductive drugs for cancer therapy: the search for tumour specificity. *Int J Radiat Oncol Biol Phys* **29**: 231–238

Adams GE, Ahmed I, Sheldon PW and Stratford IJ (1984) Radiation sensitization and chemopotentiation: RSU1069, a compound more efficient than misonidazole *in vitro* and *in vivo*. *Br J Cancer* **49**: 571–577

Baker MA, Zeman EM, Hirst VK and Brown JM (1988) Metabolisim of SR 4233 by Chinese hamster ovary cells: basis of selective hypoxic cytotoxicity. *Cancer Res* **48**: 5947–5952

Barham H, Inglis R, Chinjie EC and Stratford IJ (1996) Development and validation of a specrophotometric assay for measuring the activity of NADH:cytochrome $b_5$ reductase in human tumour cells. *Br J Cancer* **74**: 1188–1193

Belcourt MF, Hodnick WF, Rockwell S and Sartorelli AC (1996) Differential toxicity of mitomycin C and porfiromycin to aerobic and hypoxic Chinese hamster ovary cells overexpressing human NADPH:cytochrome *c* (P-450) reductase. *Proc Natl Acad Sci USA* **93**: 456–460

Biedermann KA, Wang J, Graham RP and Brown JM (1991) SR 4233 cytotoxicity and metabolism in DNA repair-competent and repair-deficient cell cultures. *r J Cancer* **63**: 358–362

Bradford M (1976) A rapid and sensitive method for quantification of microgram quantities of protein utilising the principle of protein dye binding. *Anal Biochem* **72**: 248–254

Brown JM (1993) SR4233 (Tirapazamine): a new anticancer drug exploiting hypoxia in solid tumours. *Br J Cancer* **67**: 1163–1170

Brown JM and Siim BG (1996) Hypoxia-specific cytotoxins in cancer therapy. *Semin Radiat Oncol* **6**: 22–36

Cahill A, and White INH (1990) Reductive metabolism of 3-amino-1,2,4-benzotriazine-1,4-dioxide (SR 4233) and the induction of unscheduled DNA synthesis in rat and human derived cell lines. *Carcinogenesis* **11**: 1407–1411

Dachs GU, Patterson AV, Townsend S, Adams GE, Firth JD, Ratcliffe PJ, Harris AL and Stratford IJ (1995) Testing hypoxia responsive elements for their use in cancer therapy. *Proc Am Assoc Cancer Res* **36**: 421

Denny WA, Wilson WR and Hay MP (1996) Recent developments in the design of bioreductive drugs. *Br J Cancer* **74** (suppl. 27): S32–38

Fitzsimmons SA, Lewis AD, Riley RJ and Workman P (1994) Reduction of 3-amino-1,2,4-benzotriazine-1,4-di-N-oxie (tripazamine, WIN 59075, SR 4233) to a DNA-damaging species: a direct role for NADPH:cytochrome P450 oxidoreductase. *Carcinogenesis* **15**: 1503–1510

Griffith OW (1980) Determination of glutathione and glutathione disulfide using glutathione reductase and 2-vinylpyridine. *Anal Biochem* **106**: 207–212

Hockel M, Schlenger K, Mitze M, Schaffer U and Vaupel P (1996) Hypoxia and radiation response in human tumors. *Seminars in Radiation Oncology* **6**: 3–9, 1996

Houlbrook S, Kirk J, Stuart NSA, Stratford IJ, Harris AL, Pettit GR and Carmichael J (1994) Human tumour cell lines: a valuable model for evaluating new drugs and the mechanisms underlying cytotoxic drug resistance. *Oncol (Life Sci Adv)* **13**: 69–76

Kennedy KA (1987) Hypoxic cells as specific drug targets for chemotherapy. *Anticancer Drug Res* **2**: 181–194

Keohane A, Godden J, Stratford IJ and Adams GE (1990) The effects of three bioreductive drugs (mitomycin C, RSU-1069 and SR 4233) on cell lines selected for their sensitivity to mitomycin C or ionising radiation. *Br J Cancer* **61**: 722–726

Laderoute K, Wardman P and Rauth AM (1988) Molecular mechanisms for the hypoxia-dependent activation of 3-amino-1,2,4-benzotriazine-1,4-dioxide (SR 4233). *Biochem Pharmacol* **37**: 1487–1495

Lloyd RV, Duling DR, Rumyantseva GV, Mason RP and Bridson PK (1991) Microsomal reduction of 3-amino-1,2,4-benzotriazine 1,4-dioxide to a free radical. *Mol Pharmacol* **40**: 440–445

Mason RP and Holtzman JL (1975) The mechanism of microsomal and mitochodrial nitroreductase: ESR evidence for nitroaromatic free radical intermediates. *Biochemistry* **14**: 1626–1632

Morgenstern P and Land H (1990) Advanced mammalian gene transfer: high titre retroviral vectors with multiple drug selection markers and a complementary helper-free packaging cell line. *Nucl Acid Res* **18**: 3587–3596

Mossman T (1983) Rapid colorimetric assay for cellular growth and survival: application to proliferation and cytotoxicity assays. *J Immunol Methods* **65**: 55–61

Noss MB, Panicuccir, McClelland RA and Rauth AM (1988) Preparation, toxicity and mutagenicity of 1-methyl-2-nitrosoimidazole, a toxic reduction product. *Biochem Pharmacol* **37**: 2885–2593

O'Neill P, McNeil SS and Jenkins TC (1987) Induction of DNA crosslinks *in vitro* upon reduction of the nitroimidazole-aziridines RSU-1069 and RSU-1131. *Biochem Pharmacol* **36**: 1787–1792

Osborne WR and Miler AD (1988) Design of vectors for efficient expression of human purine nucleoside phosphorylase in skin fibroblasts from enzyme-deficient humans. *Proc Natl. Acad Sci USA* **85**: 6851–6855

Patterson AV, Robertson N, Houlbrook S, Stephens MA, Adams GE, Harris AL, Stratford IJ and Carmichael J (1994) The role of DT-diaphorase in determining

© Cancer Research Campaign 1997

the sensitivity of human tumour cells to tirapazamine (SR 4233). *Int J Radiat Oncol Biol Phys* **29**: 369–372

Patterson AV, Barham HM, Chinje EC, Adams GE, Harris AL and Stratford IJ (1995) Importance of P450 reductase activity in determining sensitivity of breast tumour cells to the bioreductive drug, tirapazamine (SR4233). *Br J Cancer* **72**: 1144–1150

Peterson AJ and Prough RA (1986) Cytochrome P450 reductase and cytochrome b5 in cytochrome P450 catalysis. In *Cytochrome P450: Structure, Mechanism and Biochemistry*, Oritz de Montellano RP. (ed.) pp. 89–119. Plenum Press: New York

Plumb JA, Gerritsen M and Workman (1994) DT-diaphorase protects cells from the hypoxic cytotoxicity of the indoloquinone EO9. *Br J Cancer* **70**: 1136–1140

Riley JR and Workman P (1992) Enzymology of the reduction of the potent benzotriazene di-N-oxide hypoxic cell cytotoxin SR 4233 by NAD(P)H: (quinone acceptor) oxido-reductase (EC.1.66.99.2) purified from Walker 256 rat tumour cells. *Biochem Pharmacol* **43**: 167–174

Robertson N, Haigh A, Adams GE and Stratford IJ (1994) Factors affecting sensitivity to EO9 in rodent and human tumour cells *in vitro*: DT-diaphorase activity and hypoxia. *Eur J Cancer* **30A**: 1013–1019

Seng F and Ley K (1972) Simple synthesis of 3-amino-1,2,4 benzotriazine 1,4-dioxide. *Angew Chem Int XI*, 1009–1010

Shephard EA, Palmer CN, Segall HJ and Phillips IR (1992) Quantification of cytochrome P450 reductase gene expression in human tissues. *Arch Biochem Biophys* **294**: 168–172

Siim B, Van Zijl PL and Brown JM (1996) Tirapazamine-induced DNA damage measured using the comet assay correlates with cytotoxicity towards hypoxic tumour cells *in vitro*. *Br J Cancer* **73**: 952–960

Silver AR and O'Neil P (1986) Interaction of the aziridine moiety of RSU-1069 with nucleotides and inorganic phosphate. Implications for alkylation of DNA. *Biochem Pharmacol* **35**: 1107–1112

Stratford IJ and Stephens MA (1989) The differential hypoxic cytotoxicity of bioreductive agents determined *in vitro* by the MTT assay. *Int J Radiat Oncol Biol Phys* **16**: 973–976

Stratford IJ, Walling JM and Silver ARJ (1986*a*) The differential cytotoxicity of RSU1069: cell survival studies indicating interaction with DNA as a possible mode of action. *Br J Cancer* **53**: 339–344

Stratford IJ, O'Neill P, Sheldon PW, Silver ARJ, Walling MJ and Adams GE (1986*b*) RSU 1069, a nitroimidazole containing an aziridine group. Bioreduction greatly increases cytotoxicity under hypoxic conditions. *Biochem Pharmacol* **35**: 105–109

Van Den Hoff JB, Moorman FM and Lamers H (1992) Electroporation in 'intracellular' buffer increases cell survival. *Nucl Acid Res* **20**: 2902

Walton MI and Workman P (1987) Nitroimidazole bioreductive metabolism. Quantitations and characterisation of mouse tissue benznidazole nitroreductases *in vivo* and *in vitro*. *Biochem Pharmacol* **36**: 887–896

Walton MI and Workman P (1990) Enzymology of the reductive bioactivation of SR 4233: a novel benzotriazine di-N-oxide hypoxic cell cytotoxin. *Biochem Pharmacol* **39**: 1735–1742

Walton MI, Wolf CR and Workman P (1989) Molecular enzymology of the reductive bioactivation of hypoxic cell cytotoxins. *Int J Radiat Oncol Biol Phys* **16**: 983–986

Walton MI, Wolf CR and Workman P (1992) The role of cytochrome P450 and cytochrome P450 reductase in the reductive bioactivation of the novel benzotriazine Di-N-Oxide hypoxic cytotoxin 3-amino-1,2,4-benzotriazine-1,4-dioxide (SR 4233, WIN 59075) by mouse liver. *Biochem Pharmacol* **44**: 251–259

Wang J, Biedermann KA, Wolf CR and Crown JM (1993) Metabolism of the bioreductive cytotoxin SR 4233 by tumour cells: enzymatic studies. *Br J Cancer* **67**: 321–325

Whitmore GF and Gulyas S (1986) Studies on the toxicity of RSU-1069. *Int J Radiat Oncol Biol Phys* **12**: 1219–1222

Wilson JM, Johnston DE, Jefferson DM and Mulligan I (1988) Correction of the genetic defection hepatocytes from the Watanabe heritable hyperlipidemic rabbit. *Proc Natl Acad Sci USA* **85**: 4421–4425

Workman P and Walton MI (1989) Enzyme-directed bioreductive drug development. In *Selective activation of drugs by redox processes*, Adams G, Breccia A, Fielden EM and Wardman P (eds) pp. 89–112, Plenum Press: New York

Workman P and Stratford IJ (1993) The experimental development of bioreductive drugs and their role in cancer therapy. *Cancer Metastatis Rev* **12**: 73–82

Zeman EM, Brown JM, Lemmon MJ, Hirst VK and Lee WW (1986) SR4233: a new bioreductive agent with high selective toxicity for hypoxic mammalian cells. *Int J Radiat Oncol Biol Phys* **12**: 1239–1242

© Cancer Research Campaign 1997